# Negative impact of benign prostatic hyperplasia on fertility in dogs–a mini-review

**DOI:** 10.3389/fvets.2025.1582705

**Published:** 2025-05-09

**Authors:** Anna Domosławska-Wyderska, Sławomir Zduńczyk, Agata Rafalska

**Affiliations:** Department of Animal Reproduction with Clinic, Faculty of Veterinary Medicine, University of Warmia and Mazury in Olsztyn, Olsztyn, Poland

**Keywords:** dog, benign prostatic hyperplasia, fertility, semen quality, prostate

## Abstract

The aim of this article is to provide a comprehensive review of the negative impact of benign prostatic hyperplasia (BPH) on fertility in dogs. BPH is the most common disease of the prostate gland and one of the major causes of infertility in dogs, associated with impaired semen quality. Hormonal imbalance, oxidative stress, biochemical changes in prostatic fluid and haematospermia have been discussed as causes of reduced semen quality in dogs with BPH. Chronic prostatitis often occurs concurrently with BPH and has an additive negative effect on semen quality and fertility. In breeding dogs with infertility associated with BPH, treatment with the anti-androgen osaterone acetate or the 5α-reductase inhibitor finasteride is recommended to reduce prostate size and clinical symptoms and to restore fertility. Combined occurrence of BPH and chronic prostatitis requires simultaneous treatment of both conditions, including microbials with good penetration into the prostate. The use of antioxidants in the supportive treatment of BPH seems reasonable.

## 1 Introduction

### 1.1 Anatomy of the canine prostate

The prostate is the only accessory sex gland in the canine genital tract. The function of the prostate is to produce prostatic fluid, which accounts for approximately 90% of the volume of seminal fluid (1). The canine prostate has an oval shape with a bilobed structure that surrounds the proximal part of the urethra at the neck of the bladder and is covered by a fibromuscular capsule (2). It is located in the pelvic cavity or caudal abdomen, depending on its size, dorsal to the pubic symphysis and ventral to the rectum (3). Prostatic fluid is secreted into the urethra through several prostatic ducts. The vas deferens passes through the prostate and enters the urethra (4).

### 1.2 Benign prostatic hyperplasia

Benign prostatic hyperplasia (BPH) is one of the most important problems in intact male dogs (2, 5). It accounts for more than 50% of prostate disease cases (6, 7). BPH is defined as an increase in the overall size of the prostate caused by hyperplasia (increase in number) and hypertrophy (increase in size) of epithelial cells (3, 6). The prostate volume of affected dogs is 2 to 6.5 times greater than that of normal dogs of similar weight (8).

BPH is an age-related disease, occurring in two thirds of dogs over 5 years of age, but can also be diagnosed in young breeding males (9, 10). DeKlerk (11) reported 25% incidence of BPH in young dogs aged 1–3 years and 88% in dogs aged 5–10 years. According to Berry et al. (12) approximately 80% of sexually intact dogs over 5 years of age and 95% of dogs over 9 years of age have gross or microscopic changes associated with BPH. Canine BPH has no breed predisposition and can occur in any intact male dog (3, 8).

Initially, BPH is often asymptomatic (10). With progressive enlargement of the prostate, sanguineous discharge from the urethra, haematuria, haematospermia, defecation dysfunction and, rarely, dysuria may occur. BPH is often associated with a decrease in libido and fertility (6, 9).

The diagnosis of clinical BPH is usually based on history, clinical examination and ultrasonography. Subclinical BPH can be diagnosed by cytology using fine-needle aspiration or prostate massage or by determination of canine prostate-specific esterase (CPSE) and acid phosphatase (6). Wieszczeczyński et al. (13) proposed micro-RNA-129 (miRNA-129) and vascular endothelial growth factor (VEGF) as new useful biomarkers for the diagnosis of BPH in dogs. Recently, Laurusevičius et al. (14) developed clinical scoring systems for early detection of BPH based on non-invasive techniques such as rectal palpation, ultrasonography and CPSE analysis.

### 1.3 BPH and infertility in dogs

Infertility in male dogs, defined as the inability to produce offspring, is becoming increasingly important in clinical practice. Little is known about the causes of male infertility. They can be divided into two main groups: congenital infertility and acquired infertility. Congenital infertility is caused by genetic abnormalities and is present at birth. Acquired infertility appears after the animal has been fertile (15). BPH is considered one of the major causes of acquired infertility in male dogs (10, 15–17). One study reported that 32.8% of infertile dogs were diagnosed with BPH (18). However, the mechanisms by which BPH affects canine fertility are not fully understood.

The aim of this article is to provide a comprehensive review of the impact of BPH on fertility in male dogs.

## 2 Pathogenesis of BPH

The pathogenesis of BPH is most likely multifactorial. BPH is thought to develop primarily under the influence of the androgen metabolite dihydrotestosterone (DHT) (19, 20). With age, testosterone concentrations decrease and estrogen concentrations increase. The altered ratio of estrogen to testosterone leads to increased concentration of the androgen receptor and increased conversion of testosterone to DHT by 5-*α*-reductase in the prostate (21, 22). DTH stimulates enlargement of the canine prostate by increasing the growth of glandular epithelial cells and, to a lesser extent, stromal cells (10). BPH begins as glandular hyperplasia and progresses to cystic hyperplasia with the formation of multiple small cysts in the parenchyma (12, 20). The vascularity of the prostate tissue increases, which may lead to haemorrhagic discharge from the urethra that is not associated with urination (23, 25).

Recently, oxidative stress has been suggested to play a potential role in the pathogenesis of canine BPH (25, 26). Oxidative stress is an imbalance between the production and scavenging of reactive oxygen species (ROS) (27). There is evidence that age-related hormonal changes induce a chronic inflammatory response in the prostate, leading to the accumulation of immunocompetent cells such as macrophages and neutrophils in the prostate (28, 29). This leads to excessive generation of ROS and oxidative stress. Oxidative DNA damage induces hyperplastic transformation of prostate cells (30, 31).

## 3 Effect of BPH on fertility

BPH does not necessarily lead to infertility, but this condition is one of the leading causes of infertility in male dogs (10, 15–17). This is usually associated with a decrease in semen quality. There are relatively few studies on the effect of BPH on semen quality in dogs (Table 1).

**Table 1:** Effects of BPH on semen quality in dogs.

Effects on semen quality	Reference
Decreased sperm motility	(33)
Increased sperm DNA fragmentation and primary sperm abnormalities	(34)
Decreased sperm DNA integrity, increased mitochondrial activity and altered sperm motility patterns	(35)
Decreased percentage of fast velocity spermatozoa (RAPID), increased sperm beat cross frequency (BCF) and increased percentage of slow velocity spermatozoa (SLOW)	(36)
Decreased percentage of spermatozoa with progressive motility and in the RAPID motility subcategory and increased percentage of sperm defects	(37)
Reduced percentage of motile spermatozoa and spermatozoa with progressive motility, percentage of spermatozoa in the RAPID motility subcategory and percentage of normal spermatozoa.	(38)

Fila and Berglavaz (33) reported significant decrease in sperm motility in dogs with BPH. No significant changes in standard semen parameters were found, but a significant increase in sperm DNA defragmentation and an increased percentage of spermatozoa with primary defects formed during spermatogenesis were found in dogs with BPH compared to healthy dogs. The most common morphological defect observed was the Dag defect (circular, coiled tails) (34). Flores et al. (35) reported that BPH decreases sperm DNA integrity, increases mitochondrial activity and alters sperm motility patterns. In a study by Angrimani et al. (36), dogs with BPH showed a lower percentage of fast velocity spermatozoa (RAPID), a higher sperm beat cross frequency (BCF) and a higher percentage of slow velocity spermatozoa (SLOW) than in healthy dogs. A lower percentage of spermatozoa with progressive motility and in the RAPID motility subcategory and a higher percentage of sperm defects were observed in dogs with BPH compared to healthy dogs (37). The mean percentage of motile spermatozoa and spermatozoa with progressive motility, the percentage of spermatozoa in the RAPID motility subcategory and the percentage of normal spermatozoa were significantly lower in dogs with BPH than in healthy dogs (38). The changes described above in spermatozoa of dogs with BPH reduce their fertilizing ability and impair dog fertility.

BPH is most common in older dogs. It is generally accepted that aging has a negative effect on semen quality (37, 39). However, the decrease in semen quality in dogs with BPH is significantly greater than in healthy dogs of similar age (37, 38).

**Figure 1:**
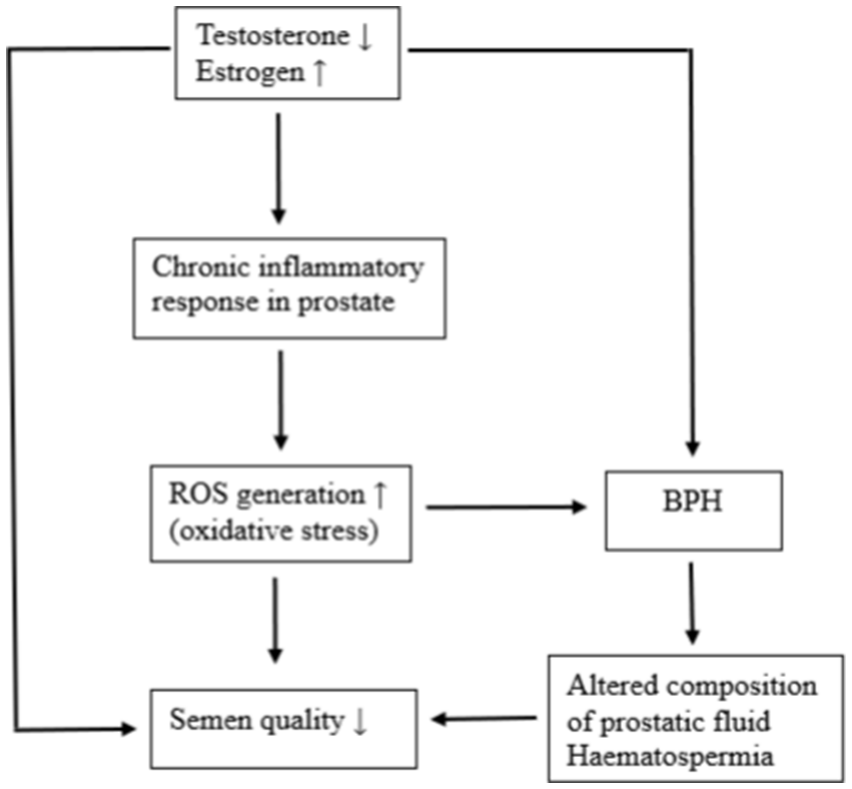
Possible mechanisms for the effect of BPH on semen quality. Reduced testosterone levels in dogs with BPH can impair spermatogenesis. A chronic inflammatory response in prostate induced by age depended hormonal changes results in the generation of large amounts of ROS. Oxidative stress in BPH dogs can cause sperm cell damage. BPH can lead to changes in the biochemical composition of the seminal plasma, resulting in reduced semen quality. The frequent presence of blood in semen of BPH dogs can adversely affect semen quality.

The mechanisms underlying the effects of BPH on semen quality are not fully understood. Hormonal imbalance, oxidative stress, biochemical changes in prostatic fluid and haematospermia have been discussed as possible causes of reduced semen quality in dogs with BPH (Figure 1).

BPH is linked to hormonal changes associated with aging. Testosterone plays an essential role in maintaining spermatogenesis (40). Lower testosterone levels have been found in dogs with BPH than in healthy dogs (32, 36, 41).

BPH is associated with oxidative stress and the accumulation of ROS can induce sperm cell damage, resulting in decreased sperm motility, velocity and morphological integrity (27, 35). Dearakhshandeh et al. (42) reported a decrease in antioxidant enzyme activities in the blood serum of dogs induced for BPH by testosterone and estradiol. Lower total serum antioxidant activity was observed in dogs with BPH compared to healthy dogs (25). A decrease in total antioxidant capacity and an increase in protein oxidation in prostatic fluid and spermatozoa were observed in dogs with BPH (43). Dogs with BPH had a higher proportion of spermatozoa producing nitric oxide, a subclass of ROS, than control dogs (38).

Prostatic fluid accounts for approximately 90% of the seminal fluid volume and provides transport and support for sperm (1). Normal prostatic fluid is clear and slightly acidic (pH 6.15–6.5) and contains various electrolytes, minerals, cholesterol, bicarbonate, fructose, lactic acid and proteins (19, 44–46). Canine prostate specific esterase accounts for more than 90% of prostate fluid proteins (44). BPH can cause changes in the biochemical composition of the seminal plasma, resulting in reduced semen quality. In dogs with BPH, a higher pH of the prostate fluid, reduced zinc and copper concentrations, and increased cholesterol concentrations have been observed (34). Ferré-Dolcet et al. (1) reported low concentrations of zinc in the prostatic fluid of dogs with BPH. In a study by Aquino-Cortez et al. (44), concentrations of glucose, cholesterol and triglycerides in prostatic fluid were higher in dogs with BPH than in healthy dogs.

Haematospermia is common in dogs with BPH. The hyperplastic gland has increased vascularity, which results in vascular leakage or hemorrhage into the gland and the blood is present in prostatic fluid and ejaculate (23). The effect of the presence of blood in semen on fertility in dogs is controversial. It is generally accepted that haematospermia may reduce sperm longevity and is associated with infertility (20, 47). However, dogs with some blood in the ejaculate may be fertile (15, 16). Rijsselaere et al. (48) showed that blood admixtures of up to 10% had no negative effect on the functional characteristics of chilled canine spermatozoa. However, blood admixtures of 2% or more in an ejaculate negatively affected semen parameters after freezing and thawing.

BPH predisposes to mainly chronic, rarely acute, bacterial infections of the prostate (3, 9, 24). Chronic bacterial prostatitis often occurs at the same time as BPH and has an additive negative effect on semen quality and fertility (10, 15, 24). It results mainly from an ascending infection from the urinary tract, and less frequently from haematogenous spread. The symptoms of chronic prostatitis are similar to those of BPH. Chronic prostatitis is often an accidental finding when infertility or recurrent cystitis are investigated (6). Diagnosis requires bacteriological and/or cytological examination of prostate fluid or prostate tissue obtained by ultrasound-guided biopsy or fine needle aspiration. Urine culture is also a reliable diagnostic tool (6, 49). The most common isolate in bacterial prostatitis is *Escherichia (E.) coli*. In addition to *E. coli*, other opportunistic bacteria ascending from the urethra such as *Staphylococcus* sp., *Pseudomonas* sp., *Klebsiella* sp., *Proteus* sp., *Enterobacter* sp., *Pasteurella* sp., *Hemophilus* sp., *Streptococcus* sp. and *Ureaplasma* sp. can cause bacterial prostatitis (49).

Chronic prostatitis has a high rate of recurrence and may progress to acute prostatitis or prostatic abscessation. Acute prostatitis can cause severe symptoms such as fever, apathy, abdominal pain, vomiting, dysuria, obstipation and unwilligness to breed. The prostate is painfull. A frequent symptom in dogs is purulent-bloody preputial discharge (4, 23, 49). In case the of an abscess the prostate is greatly enlarged and fluctuation is typical (10). The diagnosis is generally made with ultrasonography of the prostate, in combination with cytological and bacteriological examination of prostatic fluid (3, 6).

## 4 Treatment of BPH to restore fertility

There are many different methods of treating BPH in dogs. In non-breeding dogs, surgical or pharmacological castration is the treatment of choice. In breeding dogs, treatment with pharmacological agents that inhibit the production or activity of androgens is preferred in order to preserve fertility (9, 10, 24). The antiandrogen approved for the treatment of BPH in dogs is osaterone acetate (OA), which reduces the uptake of DHT in the prostate, decreases 5α-reductase activity and suppresses DHT receptor expression in prostate tissue (48). OA is available in four tablet strengths suitable for dogs weighing 3 kg to 60 kg, given orally at a dose of 0.25–0.5 mg/kg, once a day, for 7 days. Treatment with OA for 7 days rapidly reduces clinical signs and prostate volume in dogs with BPH (32, 50–52). The clinical response persists for approximately 5 months after treatment but, in some dogs, relapse of clinical signs may occur earlier (32, 51). OA had no effect on libido, but resulted in a transient decrease in seminal plasma volume, percentage of motile and progressive spermatozoa, and an increase in sperm concentration (32). Dogs treated with OA remain fertile and can still be used for mating (32, 53).

Another drug that may be considered for the treatment of BPH in breeding dogs is the 5α-reductase inhibitor finasteride, which reduces the conversion of testosterone to DHT. Treatment with finasteride at a dose of 0.2 mg/kg for 3–4 months reduced prostate size (54–56). Serum DHT concentration decreased without affecting testosterone concentration (36, 56, 57). During treatment, ejaculate volume decreased, resulting in an increase in sperm concentration (54, 55, 56). Finasteride has no effect on sperm DNA integrity (58). Fertility is not affected by treatment with finasteride (54, 55). However, finasteride is not approved in EU for use in dogs.

In the case of combined BPH and prostatitis, simultaneous treatment of both conditions is required. Treatment should also include antimicrobial agents with a good ability to penetrate prostate tissue, such as fluoroquinolones, erythromycins, clindamycin and chloramphenicol for 4–6 weeks (10, 24, 49). Prostatic abscesses require drainage and omentalization in addition to antimicrobial therapy (6, 23).

Oxidative stress plays a role in the pathogenesis of BPH and infertility. It may be increased by antioxidant deficiency. In our previous study, suboptimal Se and vitamin E status was found in male dogs with reduced fertility (59). Therefore, the supportive use of antioxidants in the treatment of BPH seems justified. Several studies have reported positive effects of selenium and vitamin E (59–61) and polyphenol (62, 63) supplementation on oxidative status and semen quality in dogs. Further studies on the usefulness of antioxidants in the supportive treatment of BPH are needed.

## 5 Conclusion

BPH is one of the major causes of infertility in male dogs. It is associated with reduced semen quality. Hormonal imbalance, oxidative stress, biochemical changes in prostatic fluid and haematospermia may contribute to this decline. Chronic bacterial prostatitis often co-occurs with BPH and has an additive negative effect on semen quality and fertility. In breeding dogs with infertility following BPH, treatment with the anti-androgen osaterone acetate or the 5α-reductase inhibitor finasteride is recommended to restore fertility. In the case of combined BPH and prostatitis, treatment should also include antimicrobial agents with a good ability to penetrate prostate tissue. The adjunctive use of antioxidants in the treatment of BPH seems justified.
